# Inhibition of apoptosis through AKT-mTOR pathway in ovarian cancer and renal cancer

**DOI:** 10.18632/aging.204564

**Published:** 2023-02-27

**Authors:** Hongrun Chen, Lianfeng Zhang, Meini Zuo, Xiaowen Lou, Bin Liu, Taozhu Fu

**Affiliations:** 1 Department of Urology, China Aerospace Science and Industry Corporation 731 Hospital, Beijing 100074, China; 2 Department of Social Work, The First People's Hospital of Fuyang District of Hangzhou, Hangzhou 311400, Zhejiang, China; 3 Department of Urology, The Fourth Hospital of Hebei Medical University, Shijiazhuang 050011, Hebei, China

**Keywords:** Akt-mTOR pathway, renal cancer, ovarian cancer, bioinformatics, verification, biomarker

## Abstract

Objective: Ovarian cancer and renal cancer are malignant tumors; however, the relationship between TTK Protein Kinase (TTK), AKT-mTOR pathway and ovarian cancer, renal cancer remains unclear.

Methods: Download GSE36668 and GSE69428 from Gene Expression Omnibus (GEO) database. Weighted gene co-expression network analysis (WGCNA) was performed. Created protein-protein interaction (PPI) network. Used Gene Ontology analysis (GO) and Kyoto Encyclopedia of Genes and Genomes (KEGG) for functional enrichment analysis. Gene Set Enrichment Analysis (GSEA) analysis and survival analysis were performed. Created animal model for western blot analysis. Gene Expression Profiling Interactive Analysis (GEPIA) was performed to explore the role of TTK on the overall survival of renal cancer.

Results: GO showed that DEGs were enriched in anion and small molecule binding, and DNA methylation. KEGG analysis presented that they mostly enriched in cholesterol metabolism, type 1 diabetes, sphingolipid metabolism, ABC transporters, etc., TTK, mTOR, p-mTOR, AKT, p-AKT, 4EBP1, p-4EBP1 and Bcl-2 are highly expressed in ovarian cancer, Bax, Caspase3 are lowly expressed in ovarian cancer, cell apoptosis is inhibited, leading to deterioration of ovarian cancer. Furthermore, the TTK was not only the hub biomarker of ovarian cancer, but also one significant hub gene of renal cancer, and its expression was up-regulated in the renal cancer. Compared with the renal cancer patients with low expression of TTK, the patients with high expression of TTK have the poor overall survival (*P* = 0.0021).

Conclusion: TTK inhibits apoptosis through AKT-mTOR pathway, worsening ovarian cancer. And TTK was also one significant hub biomarker of renal cancer.

## INTRODUCTION

Ovarian cancer and renal cancer can occur at any age, its incidence is increasing [1, 2]. It initially manifests with very little symptoms and is difficult to detect at first. When a patient presents their symptoms to a hospital for examination, it is often in late stages, and treatment has already been delayed [3]. Ovarian and renal cancers pose a major health threat to women. Ovarian cancer and renal cancer can seriously affect the life of patients, and even threaten their lives. Surgical treatment is the main treatment, supplemented by chemotherapy, radiotherapy, immunotherapy, etc., [4, 5]. However, pathogenesis of ovarian cancer and renal cancer is unknown, and more researches are needed.

Bioinformatics is the intersection of biology and computer science. It is also an important content of proteome research [6]. Today bioinformatics technology is advancing, it has been used to analyze known or new gene products [7].

TTK (TTK Protein Kinase) is a Protein coding gene located in chromosome 6q13-6q21. Its related pathways include DNA damage and cell cycle, encodes phosphorylated Protein kinases on serine [8]. TTK protein kinase is a mitotic kinase that participates in control of cell progression through mitosis, and affects cell division [9].

Akt/mTOR pathway has multiple initiation mechanisms, and it manifests in some cancer subtypes [10]. Akt/mTOR pathway affects protein translation, survival, metabolism, its abnormalities can cause cancer [11]. But, how TTK and AKT-mTOR pathways affect ovarian cancer and renal cancer is uncertain.

This research with aid of bioinformatics, digging at core genes of ovarian cancer and renal cancer, through some experiments to determine whether TTK and AKT-mTOR pathway can affect ovarian cancer and renal cancer.

## METHODS

### Ovarian cancer data set

Profiles of ovarian cancer GSE36668 and GSE69428 were generated using GPL570, at the same time we also use GSE140082 data set as a survival data validation from GEO database (http://www.ncbi.nlm.nih.gov/geo/). Among them, GSE36668 included 8 ovarian cancer and 4 normal tissue samples, GSE69428 included 10 ovarian cancer and 10 normal tissue samples to get DEGs in ovarian cancer.

### Batch processing

For combination of multiple data sets, we firstly combined data sets GSE36668 and GSE69428 with R software package inSilicoMerging (https://doi.org/10.1186/1471-2105-13-335) to obtain merge matrix. Further, we used remove Batch Effect function of the R software package limma (version 3.42.2) to remove batch effect, finally obtained matrix after removing batch effect, which was applied to subsequent analysis.

### Screening of DEGs

R package “limma” was used for probe summary and background correction of batched-effect post-matrix for GSE36668 and GSE69428. Used Benjamini-Hochberg method to set raw *P* values. Used fold change (FC) to get false discovery rate (FDR). Cut-off criterion for DEG was FDR < 0.05. And make a volcano diagram.

### Weighted gene co-expression network analysis (WGCNA)

Top 50% genes with smallest median absolute deviation were acquired and excluded. For all genes in pairs perform Pearson correlation matrix and average chain method, using power function a|mn=| C|mn |^β build weighted adjacency matrix. After choose soft threshold parameter, converts adjacency matrix to topological overlap matrix. Average linkage hierarchical clustering was performed, minimum size (genome) was 30. Sensitivity was set to 3. We calculated the phase divergence of module feature genes, incorporating modules with distances less than 0.25. At the same time, we also predicted the inter-relationship of genes in the module to obtain core genes.

### PPI network

Intersect the core genes of WGCNA with the genes selected in the volcano map. The list of genes was input into the STRING (http://string-db.org/) database to build a PPI network (confidence >0.4) for predicting core genes. PPI network was imported into cytoscape software. Three algorithms (MCC, MNC, DMNC) were used to calculate ten best correlation genes and take intersection, and core gene list was exported after visualization.

### Functional enrichment analysis

Gene Ontology analysis (GO) and Kyoto Encyclopedia of Genes and Genomes (KEGG) analysis are computational methods for assessing gene function and biological pathways. The core of this research will figure out Venn diagram list input KEGG rest API (https://www.kegg.jp/kegg/rest/keggapi.html), to obtain the latest KEGG Pathway gene annotation. As the background, the genes were mapped to the background set, and the R software package clusterProfiler (version 3.14.3) was used for enrichment analysis to obtain the results of gene set enrichment. Also use R software package org.Hs.eg.db (version 3.1.0) gene in the GO annotation, as the background, to map genes to background in the collection, set the minimum gene sets 5, biggest gene sets, 5000, *P* value of < 0.05 and a FDR of < 0.25 were considered as measures of statistical significance.

This study will Wayne figure out the difference of gene list input KEGG rest API (https://www.kegg.jp/kegg/rest/keggapi.html) to get latest KEGG Pathway gene annotation, Used R package clusterProfiler (version 3.14.3) for enrichment analysis to get results of gene set enrichment. GO annotation of genes in R software package org.Hs.eg.db (version 3.1.0) was used as background, genes were mapped to background set. The minimum gene set was 5, maximum gene set was 5000. *P* value of < 0.05, FDR of < 0.25 were measures of statistical significance.

In addition, we use Metascape database (http://metascape.org/gp/index.html), for above differences in gene enrichment of function analysis and export list.

### GSEA analysis

GSEA, computational method that can perform GO and KEGG analyses on complete genomes. In our study, we grouped the samples by tumor tissue and normal tissue, performed GO and KEGG analyses on the whole genome. Developed by GSEA.

### Heat map of gene expression

By R package heatmap to make a heatmap of expression degree of core genes found by three algorithms in PPI network to visually displayed expression differences of core genes between cancer and normal tissue.

### Survival analysis

We selected the ovarian cancer survival data from the dataset GSE140082, used R software package maxstat (version:0.7–25) to calculate optimal cut-off value of RiskScore of ten core genes, best cut-off value is calculated, Survfit function of the R package survival was further used to find prognostic differences. We also used R package forest to make a forest map of 10 core genes to observe whether each independent core gene had a significant effect on prognosis of renal cancer.

### Establishment of animal models

Measure weight of C57BL/6J mice (Female, 8 ± 1 Weeks) and recorded. They were then randomly numbered and grouped. Divide rats into 4 groups of 6 rats each. Group A: Con; Group B: OV; Group C: OV/TTK-OE; Group D: OV/TTK-KO. The oncogene of human ovarian cancer tumor was directly transferred into mice for expression. The target gene (genome fragment) was injected into the fertilized egg of the mouse by microinjection method, and the fertilized egg was implanted into the fallopian tube (or uterus) of the recipient animal to develop transgenic mice carrying foreign gene.

### Western blot

Extracting total protein from tissue, after concentration was determined by UV method, 1/4 of protein sample volume of 5× protein loading buffer (reduced) was added to tissue, boiled at 100°C for 10 min, cooled, packed and frozen in −80°C refrigerator until use. Protein samples were subjected to 12% SDS-PAGE gel electrophoresis, membrane transformation, other operations. Block 5% skim milk at room temperature for 1 h. Added primary antibody, incubated samples overnight at 4°C. After shaking TBST for 3 times (5 min/time), rabbit secondary antibody was added. After incubation for 1 h at room temperature, TBST was shaken 3 times (5 min/time). Analyzed results after chemiluminescence solution was developed.

### GEPIA for the TTK and renal cancer

Through the GEPIA, the expression of TTK in the renal cancer was analyzed, and the relationship between relative expression of TTK and pathological stage was also explored. Furthermore, the overall survival of renal cancer was analyzed.

### Data availability

The datasets generated during and/or analyzed during the current study are available from the corresponding author on reasonable request.

## RESULTS

### Differentially expressed genes (DEGs)

**Figure 1:**
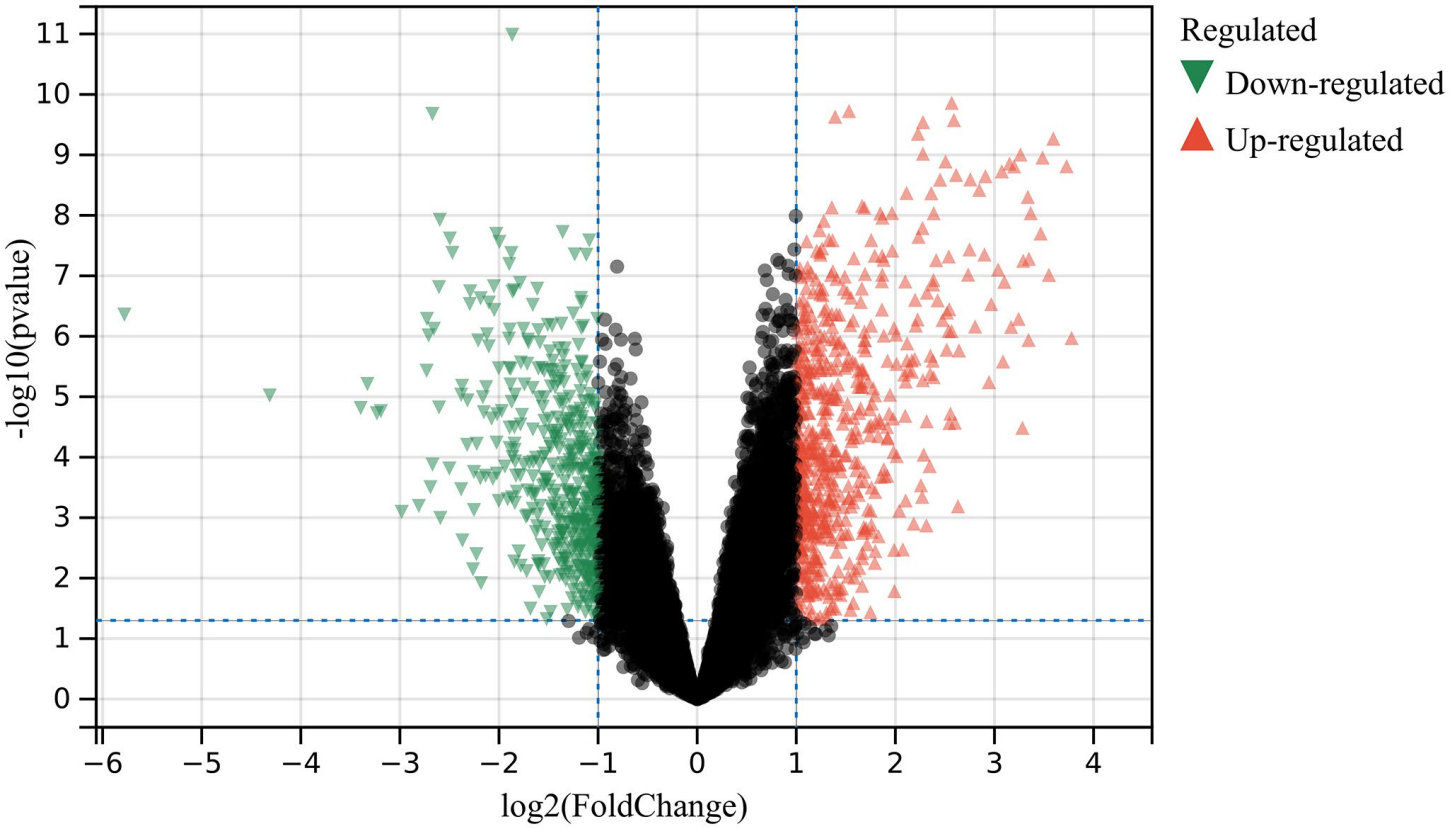
**DEGs were identified.** 602 up-regulated (Red) and 450 down-regulated genes (Green).

1052 DEGs were found based on DEGs identified in debatching merge matrix of GSE36668 and GSE69428 (Figure 1).

### WGCNA analysis

**Figure 2:**
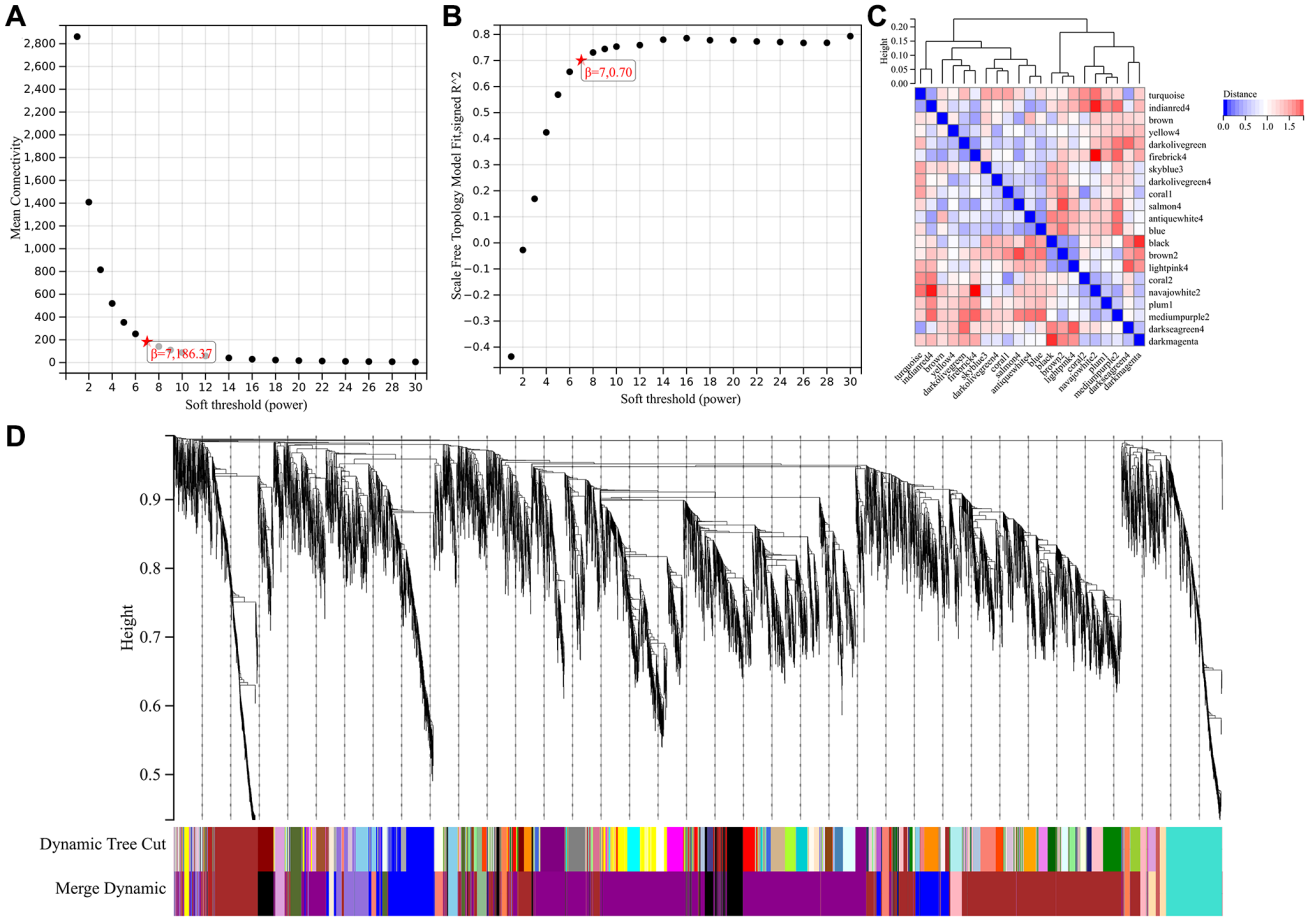
**WGCNA analysis.** (**A**) β = 7,186.37; (**B**) β = 7,0.707; (**C**) 14 important modules; (**D**) A high degree of independence between modules.

**Figure 3:**
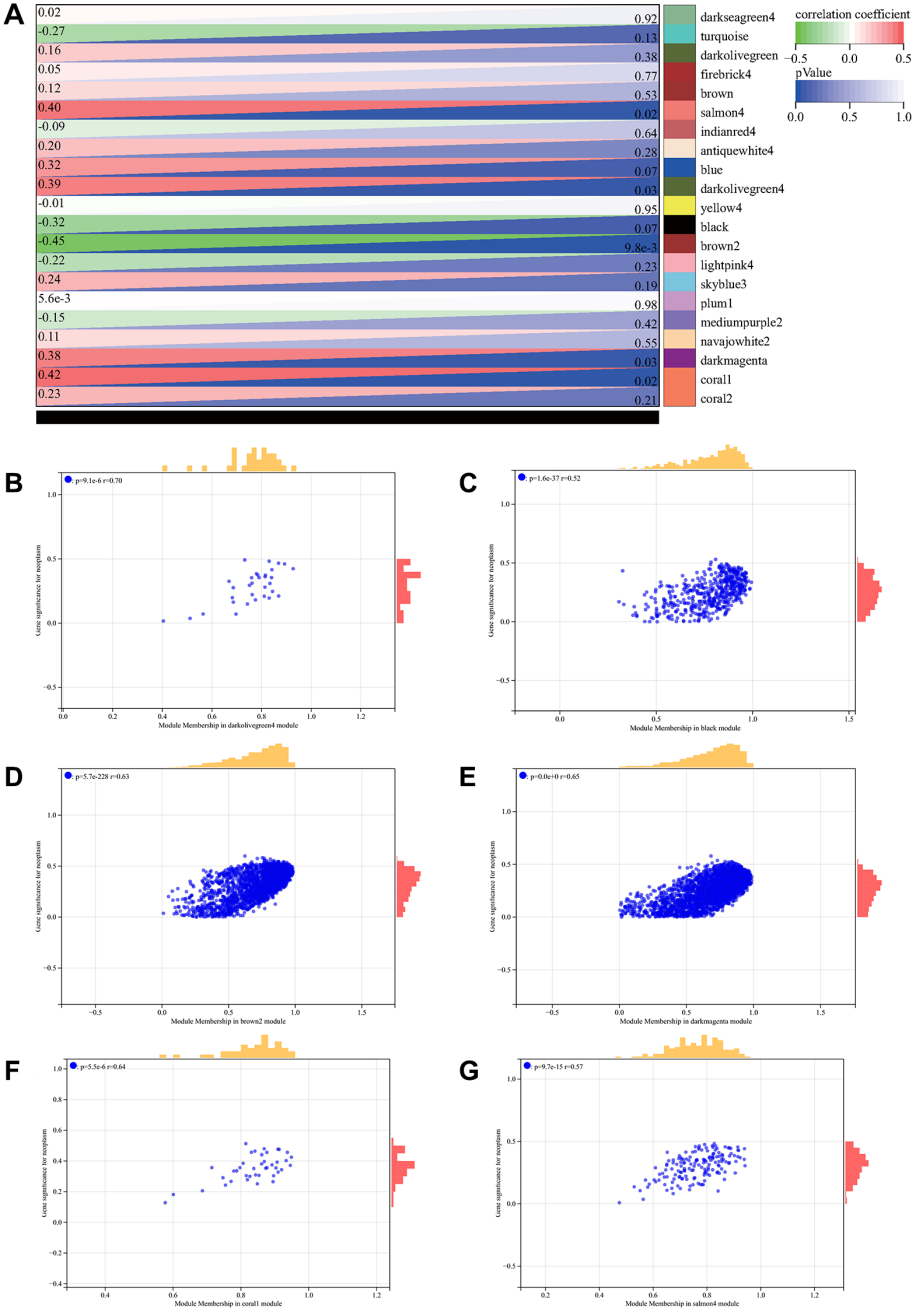
**WGCNA analysis.** (**A**) relationship between module and clinical manifestations of ovarian cancer; (**B**) Module Membership in darkolivegreen4 module: *p* = 9.1e-6, r = 0.70; (**C**) Module Membership in black module: *p* = 1.6e-37, r = 0.52; (**D**) Module Membership in brown2 module: *p* = 5.7e-228, r = 0.63; (**E**) Module Membership in darkmagenta module: *p* = 0.0e + 0, r = 0.65; (**F**) Membership in coral1 module: *p* = 5.5e-6, r = 0.64; (**G**) Membership in salmon4 module: *p* = 9.7e-15, r = 0.57.

Soft threshold power in WGCNA analysis was set to 7, which is lowest power for scale-free topological fit index of 0.9 (Figure 2A, 2B). Hierarchical clustering trees were constructed for all genes and yielded 14 significant modules (Figure 2C). Interactions between these modules were then analyzed (Figure 2D). Relationship between modules and clinical manifestations of ovarian cancer is shown in Figure 3A. Highly correlated modules were then plotted against a column scatter plot of clinical characteristics (Figure 3B–3G). We take as standard | MM | > 0.8, a total of 1909 in clinical important modules with high connectivity genes have been identified as core.

### PPI network

**Figure 4:**
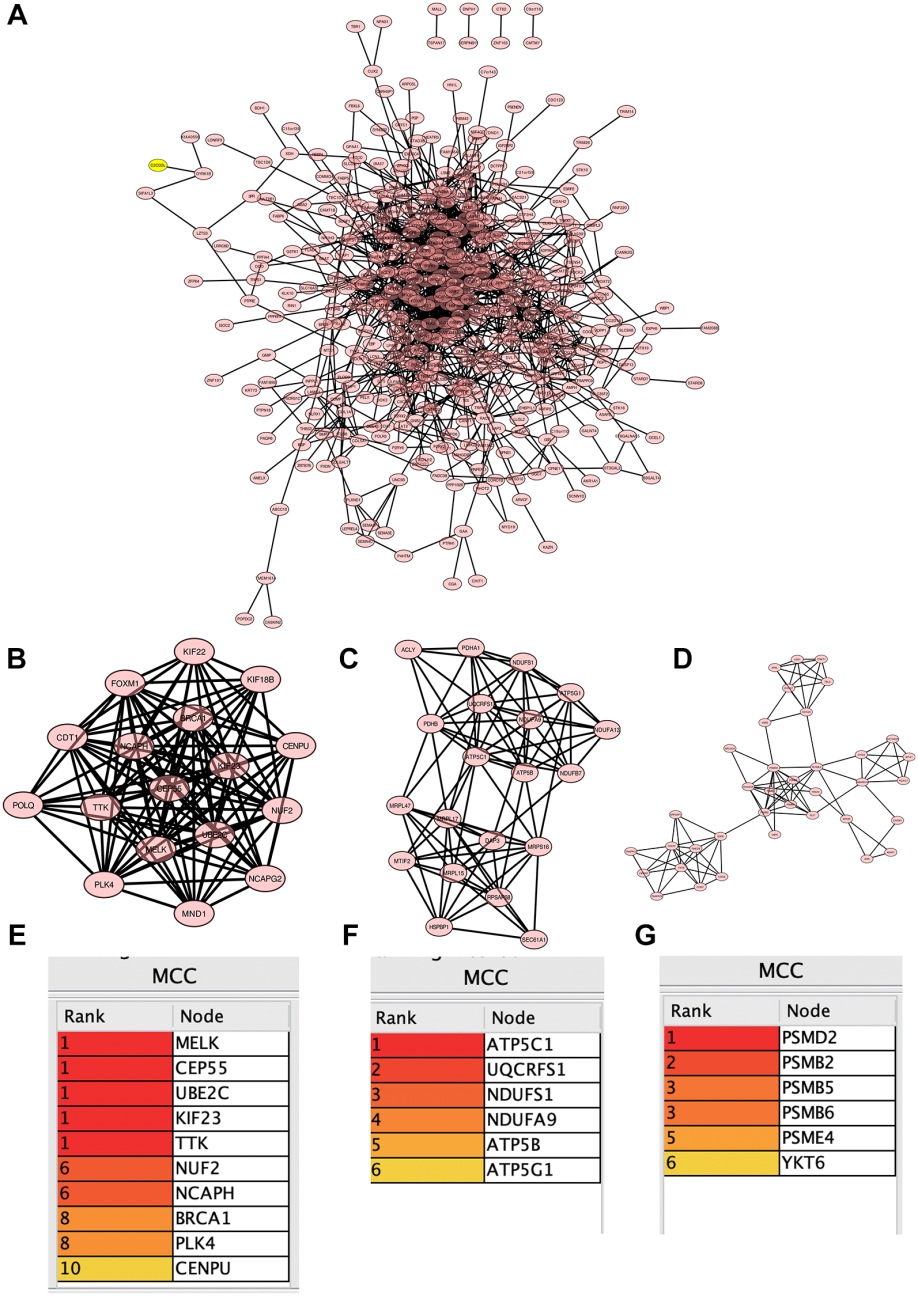
**Construction of protein-protein interaction (PPI) network.** (**A**) PPI network; (**B**–**D**) 3 core modules; (**E**–**G**) 22 common central genes.

Selected DEGs in tumor group, analyzed by Cytoscape software (Figure 4A), a total of 3 core modules were obtained using MCODE algorithm (Figure 4B–4D), and 22 common hub genes were obtained using MCC algorithm to identify the core genes (Figure 4E–4G).

### Functional enrichment analysis

**Figure 5:**
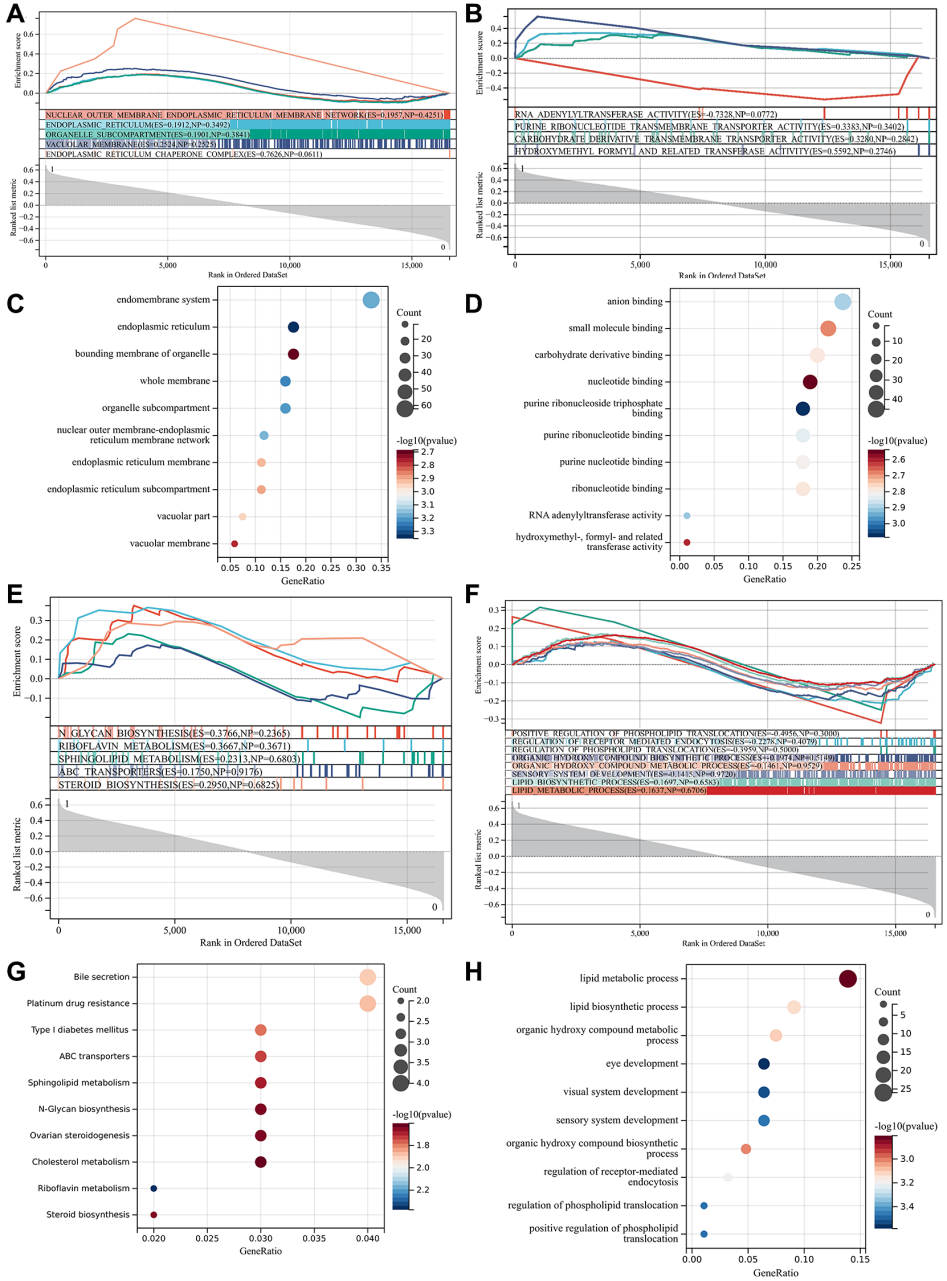
**Functional enrichment analysis and GSEA analysis.** (**A**) GO and KEGG enrichment projects were verified; (**B**) GO and KEGG enrichment projects were verified; (**C**) GO enrichment term; (**D**) GO enrichment term; (**E**) GO enrichment terms under GSEA analysis; (**F**) KEGG enrichment terms under GSEA analysis; (**G**) GO enrichment term; (**H**) KEGG enrichment item.

Used GSE10540 gene matrix for enrichment analysis, and it can be seen that the GSEA enrichment project was validated with the GO and KEGG enrichment projects among differentially expressed genes, which were mainly enriched in the endoplasmic reticulum, organelle subcompartment, purine ribonucleotide transmembrane transporter (Figure 5A, 5B).

In GO analysis, they were mostly concentrated in anion and small molecule binding, eye development, visual system formation, lipid biosynthesis, sensory system formation, DNA methylation. In KEGG analysis, target genes were mostly concentrated in cholesterol metabolism, type 1 diabetes, sphingolipid metabolism, ABC transporters, etc. Figure 5C, 5D are bubble plot *P*-values of GO-enriched terms. GO enrichment terms under GSEA analysis are shown in Figure 5E, 5F are KEGG enrichment terms. At the aspect of biological process, the DEGs were mainly enriched in the bile secretion, ovarian steroidogenesis, steroid biosynthesis (Figure 5G, 5H). Are bubble plot *P*-values of KEGG-enriched terms.

### Metascape enrichment analysis

**Figure 6:**
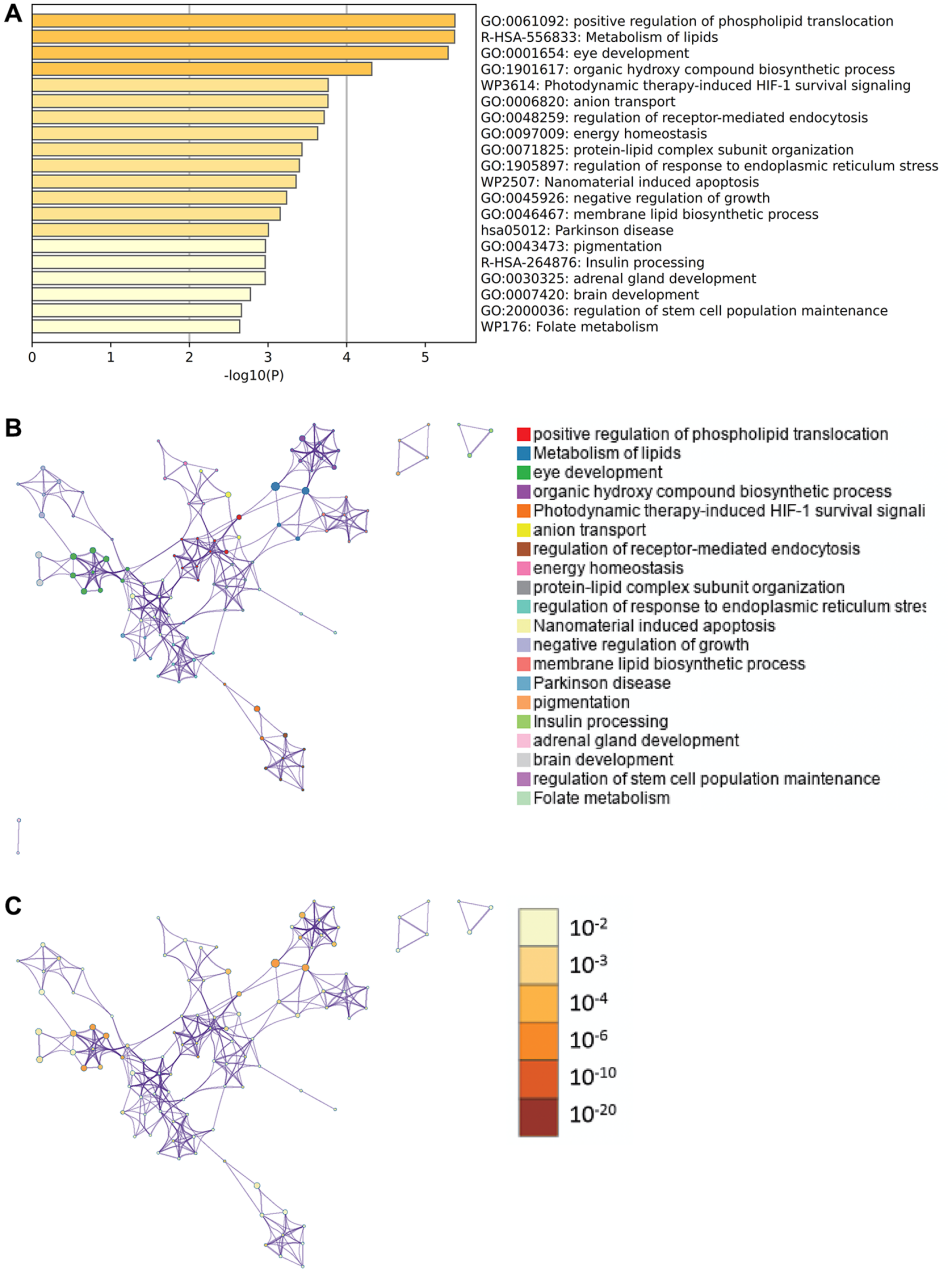
**Metascape enrichment analysis.** (**A**) Enrichment of GO; (**B**) Enrichment networks colored by enrichment terms; (**C**) Enrichment networks colored by *P*-value.

**Figure 7:**
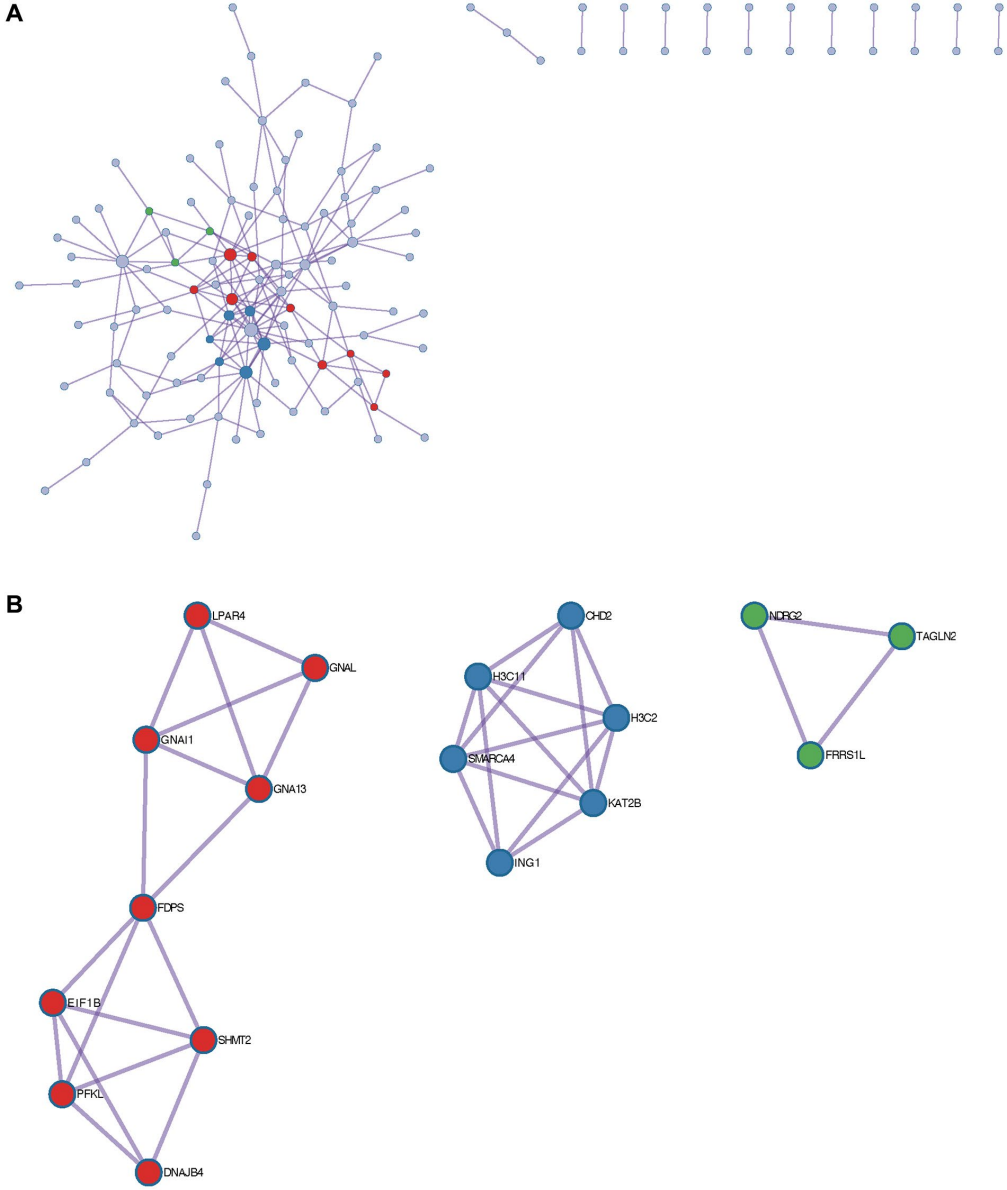
**Metascape enrichment analysis.** (**A**) PPI network. (**B**) 3 core modules.

Content enriched by Metascape includes GO enrichment terms (Figure 6A), enrichment networks colored by enrichment terms and *P* values (Figure 6B, 6C), and PPI networks and core modules formed in the Metascape website based on core genes (Figure 7A, 7B).

### Survival analysis

**Figure 8:**
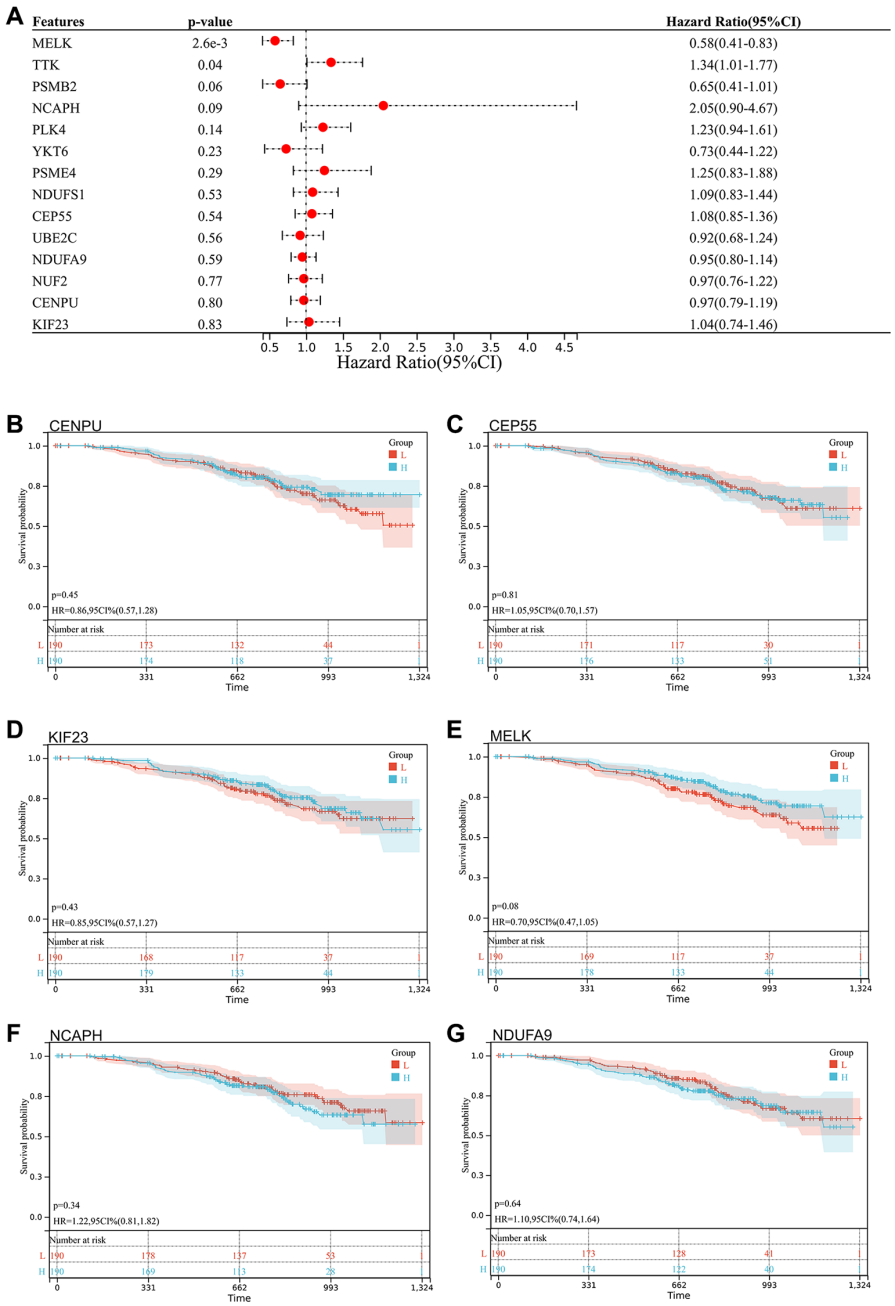
**Survival analysis, survivorship curve.** (**A**) Survival data of 14 core genes with significantly different expression and GSE140082; (**B**) CENPU: *p* = 0.45, HR = 0.86, 95% CI (0.57, 1.28); (**C**) CEP55: *p* = 0.81, HR = 1.05, 95% CI (0.70, 1.57); (**D**) KIF23: *p* = 0.43, HR = 0.85, 95% CI (0.57, 1.27); (**E**) MELK: *p* = 0.08, HR = 0.70, 95% CI (0.47, 1.05); (**F**) NCAPH: *p* = 0.34, HR = 1.22, 95% CI (0.81, 1.82); (**G**) NDUFA9: *p* = 0.64, HR = 1.10, 95% CI (0.74, 1.64).

**Figure 9:**
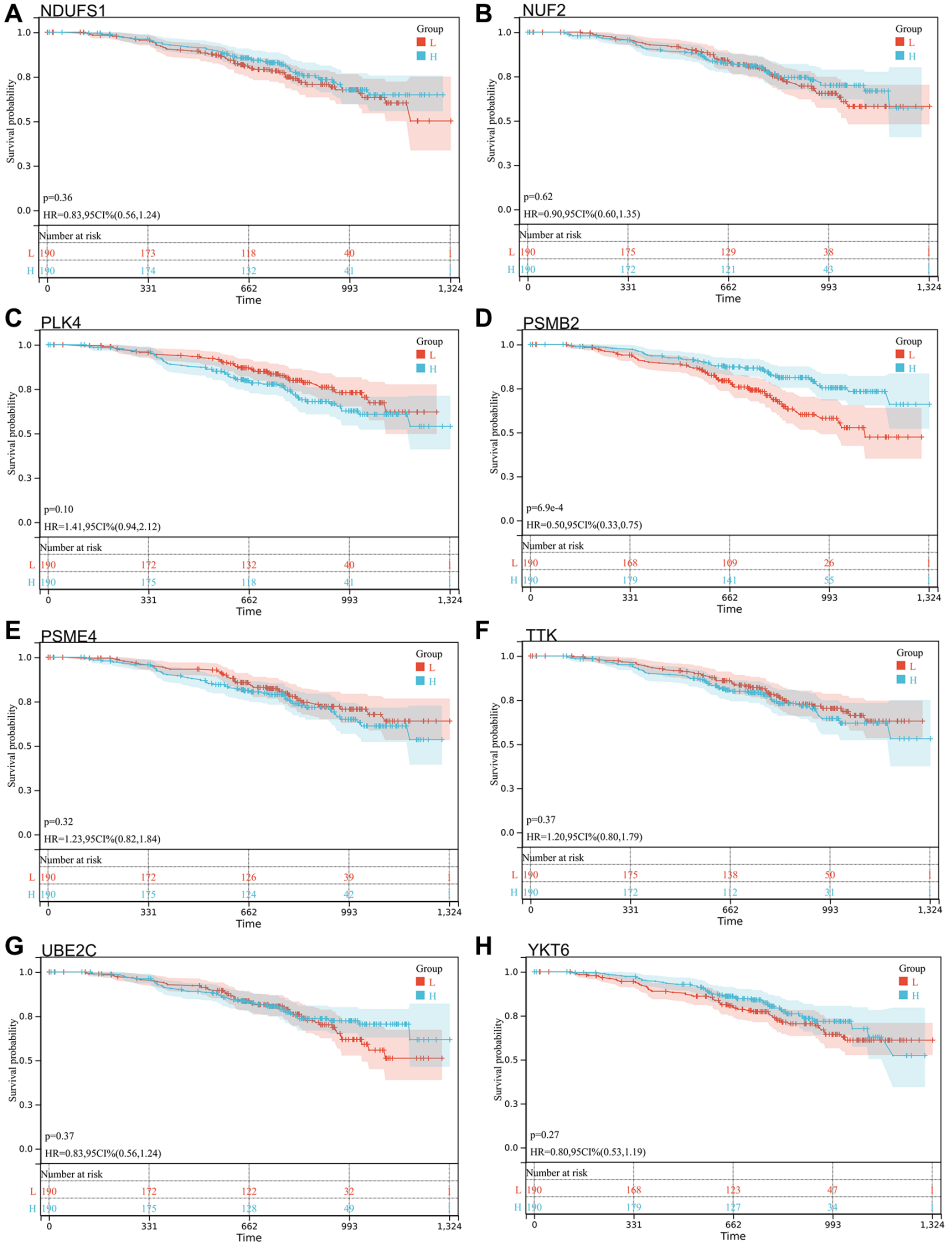
**Survival analysis, forest map.** (**A**) NDUFS1: *p* = 0.36, HR = 0.83, 95% CI (0.56, 1.24); (**B**) NUF2: *p* = 0.62, HR = 0.90, 95% CI (0.60, 1.35); (**C**) PLK4: *p* = 0.10, HR = 1.41, 95% CI (0.94, 2.12); (**D**) PSMB2: *p* = 6.9e-4, HR = 0.50, 95% CI (0.33, 0.75); (**E**) PSME4: *p* = 0.32, HR = 1.23, 95% CI (0.82, 1.84); (**F**) TTK: *p* = 0.37, HR = 1.20, 95% CI (0.80, 1.79); (**G**) UBE2C: *p* = 0.37, HR = 0.83, 95% CI (0.56, 1.24); (**H**) YKT6: *p* = 0.27, HR = 0.80, 95% CI (0.53, 1.19).

We selected 14 core genes with large expression differences to combine KM survival curve and forest plot with the survival data of GSE140082, and MELK and TTK genes had significant prognostic differences (*P* < 0.05. Figures 8, 9).

### CTD analysis

**Figure 10:**
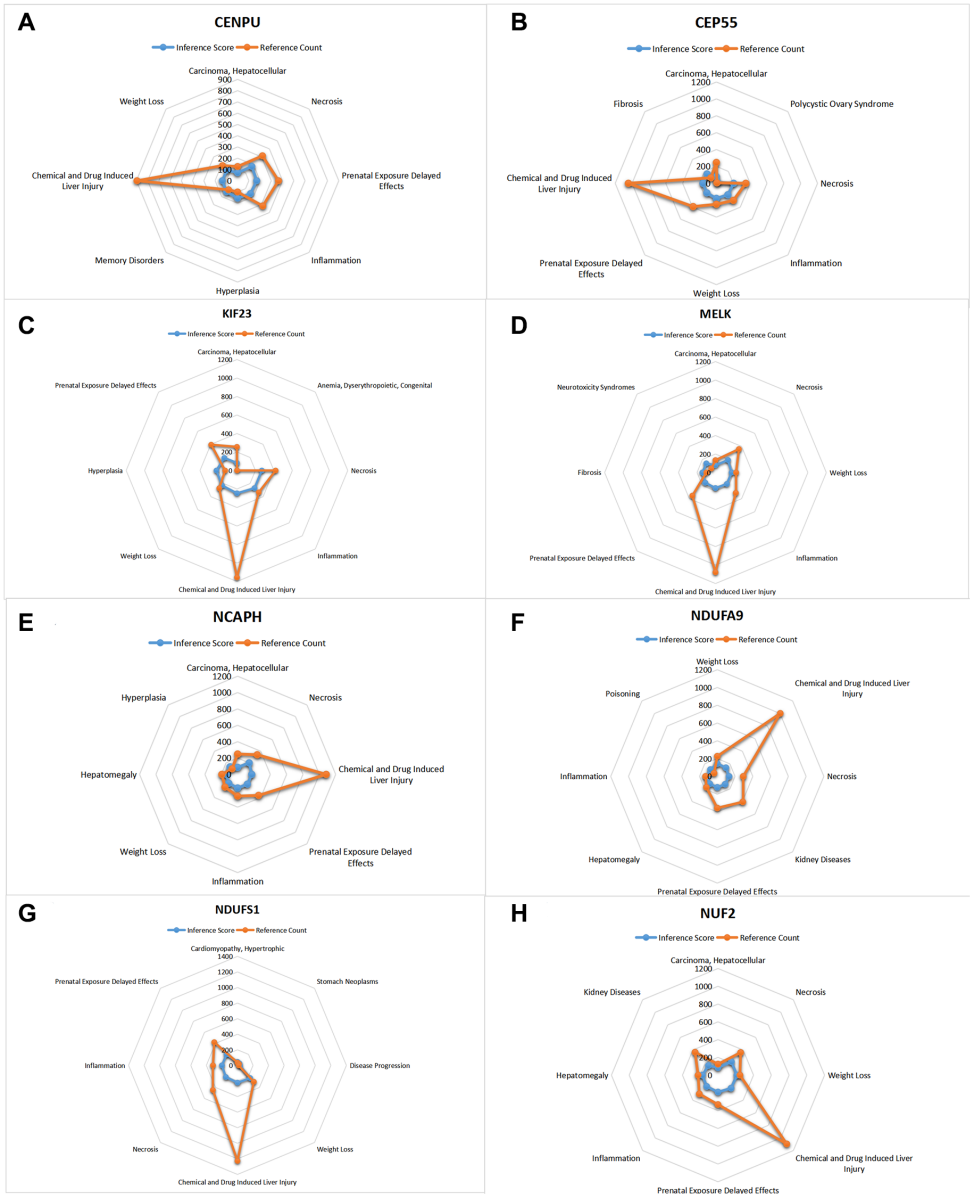
**CTD analysis.** The list of core genes was entered into the CTD website. (**A**) CENPU; (**B**) CEP55; (**C**) KIF23; (**D**) MELK; (**E**) NCAPH; (**F**) NDUFA9; (**G**) NDUFS1; (**H**) NUFE.

**Figure 11:**
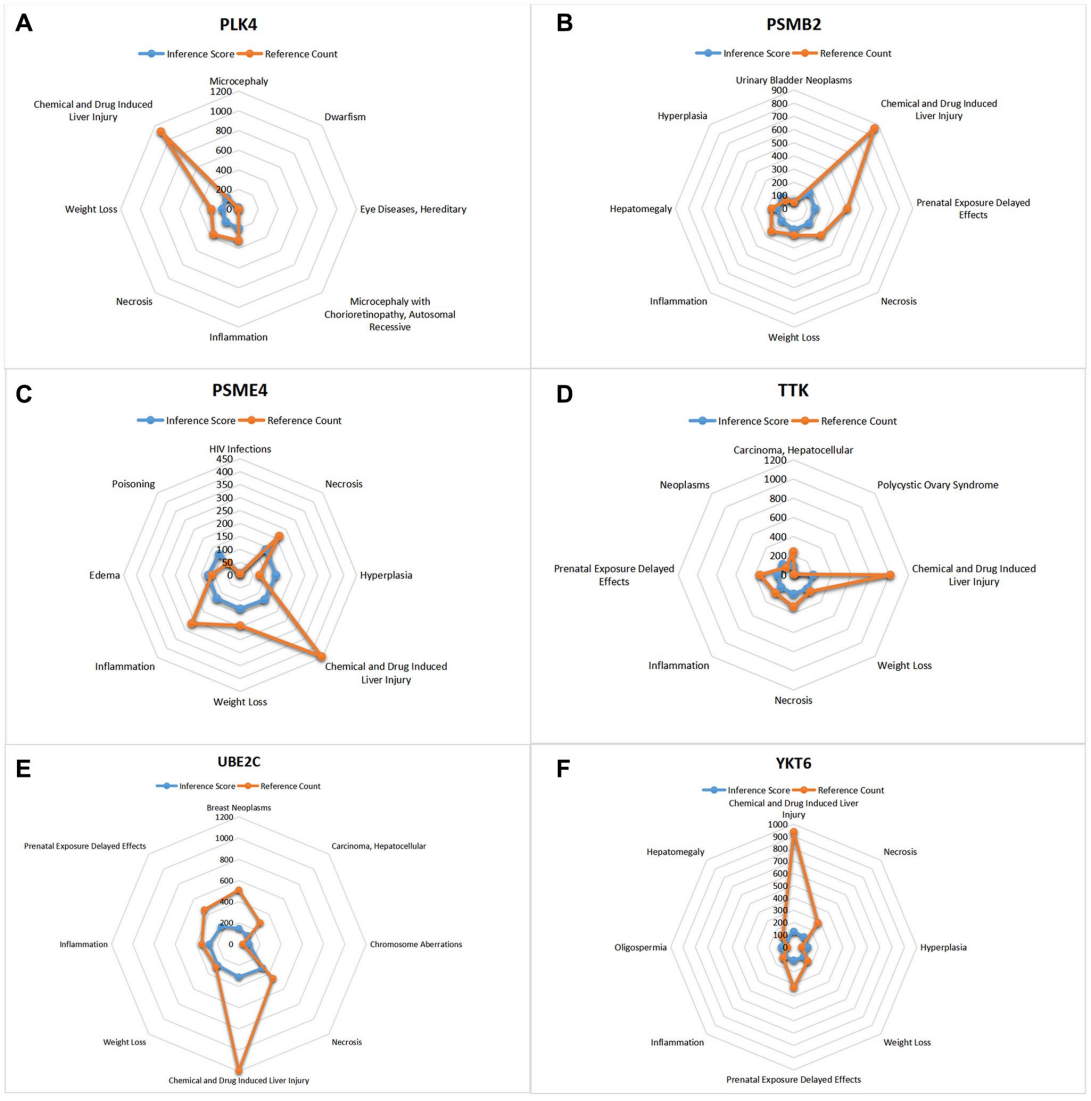
**CTD analysis.** (**A**) PLK4; (**B**) PSMB2; (**C**) PSME4; (**D**) TTK; (**E**) UBE2C; (**F**) YKT6.

In this study, we input the core gene list into the CTD website to search for diseases associated with core genes and improve the understanding of gene-disease association (Figures 10, 11).

### microRNAs analysis

In this study, we input the hub gene list into TargetScan to find relevant miRNA and improve the understanding of gene expression regulation (Table 1).

**Table 1:** A summary of miRNAs that regulate hub genes.

	**Gene**	**MIRNA**		
1	PSMB2	hsa-miR-31-5p		
2	PSME4	hsa-miR-6088	hsa-miR-143-3p	hsa-miR-4770
3	YKT6	hsa-miR-129-1-3p	hsa-miR-129-2-3p	
4	NCAPH	hsa-miR-493-5p		
5	NDUFS1	hsa-miR-599	hsa-miR-320d	hsa-miR-320a
6	KIF23	hsa-miR-103a-3p	hsa-miR-107	
7	MELK	hsa-miR-802		
8	TTK	hsa-miR-455-3p.1		
9	CEP55	hsa-miR-144-3p		
10	NUF2	hsa-miR-599		
11	NDUFA9	none		
12	PLK4	none		
13	CENPU	none		
14	UBE2C	none		

### Heat map

**Figure 12:**
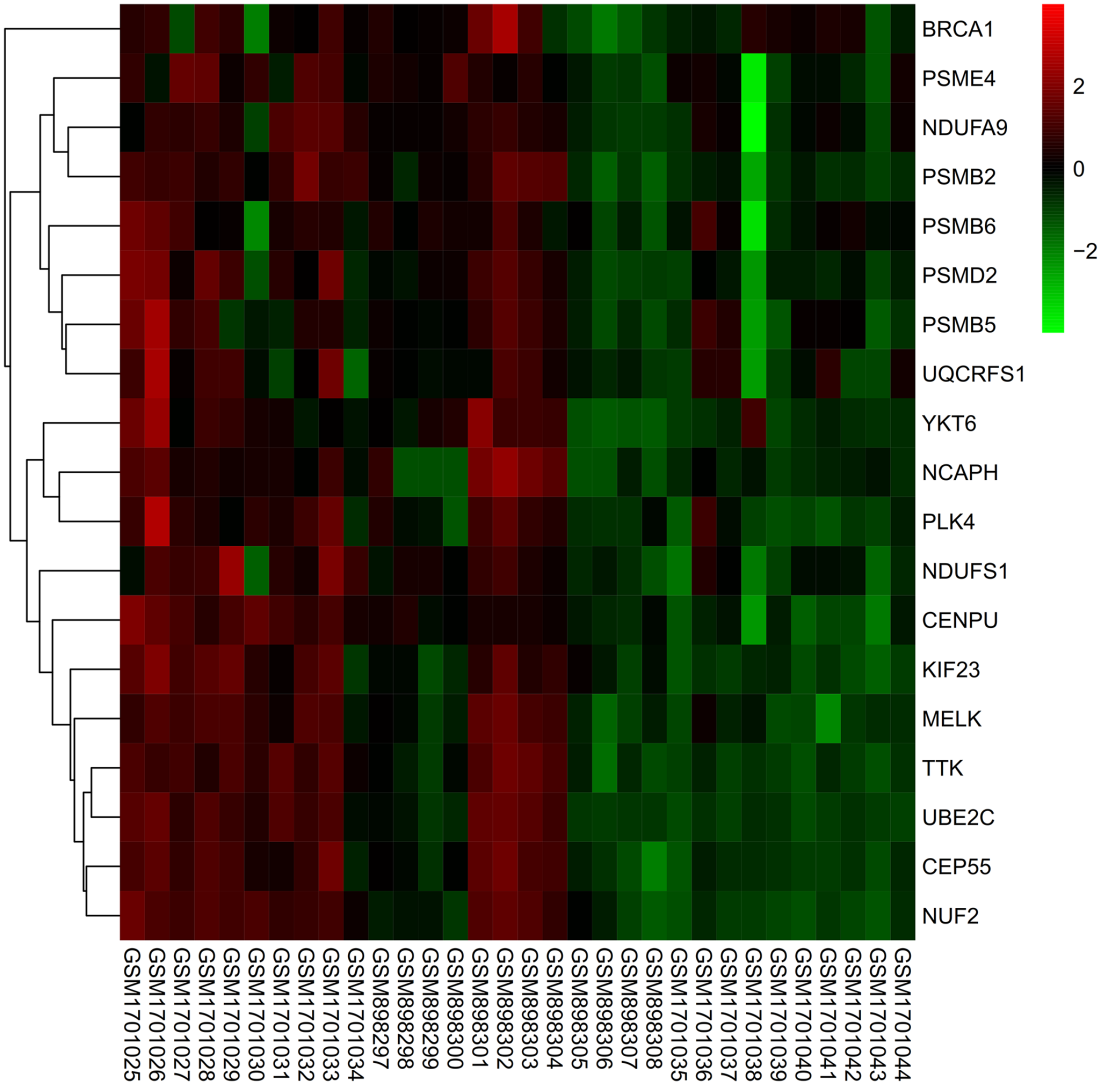
**Heat map.** The expression levels of all genes were up-regulated in the tumor group.

Expression of core genes in de-batched matrix was subjected to heat map processing, expression of all genes was up-regulated in tumor group (Figure 12).

### Western blot (WB)

**Figure 13:**
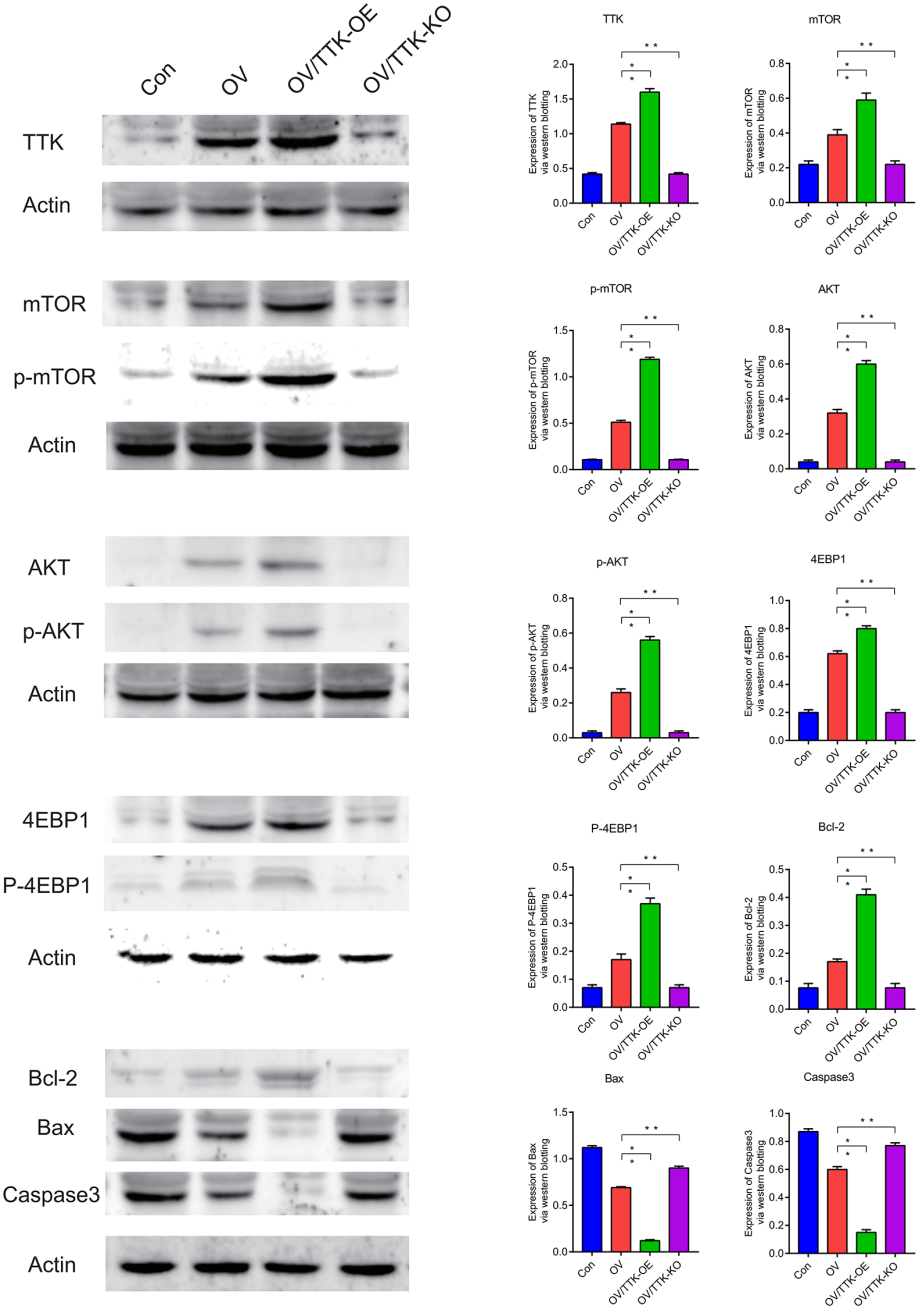
**Western blotting.** TTK, mTOR, p-mTOR, AKT, p-AKT, 4EBP1, P-4EBP1, Bcl-2, Bax, Caspase3. *P* < 0.05.

Western blotting analysis showed that TTK, mTOR, AKT were highly expressed in ovarian cancer (*P* < 0.05). TTK, mTOR, p-mTOR, AKT, p-AKT, 4EBP1, p-4EBP1, Bcl-2 are highly expressed in ovarian cancer, Bax, Caspase3 are lowly expressed in ovarian cancer, and cell apoptosis is inhibited, leading to the deterioration of ovarian cancer. When TTK, mTOR and AKT were overexpressed, main molecules of apoptotic pathway were more inhibited. Conversely, main molecules of the apoptotic pathway are activated to induce apoptosis in ovarian cancer cells (Figure 13).

### Role of TTK on the renal cancer

**Figure 14:**
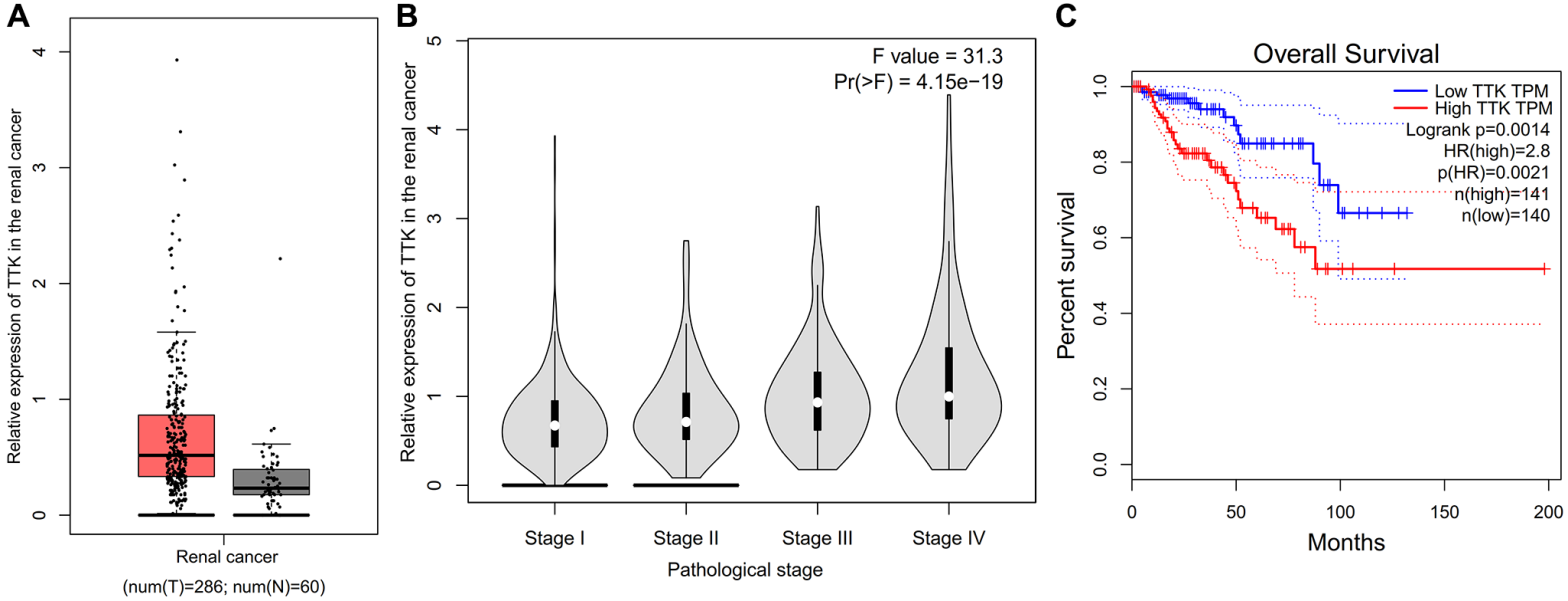
**Role of TTK on the renal cancer.** (**A**) Comparison of expression of TTK between normal and renal cancer. (**B**) Correlation between expression of TTK and the pathological stage of renal cancer. (**C**) Overall survival of renal cancer.

Compared with the normal tissues, the expression of TTK in the renal cancer was higher (Figure 14A). There is a positive correlation between expression of TTK and the pathological stage of renal cancer (*P* < 0.05, Figure 14B). Compared with the renal cancer patients with low expression of TTK, the patients with high expression of TTK have the poor overall survival (*P* = 0.0021, Figure 14C).

## DISCUSSION

Patients with high expression of TTK in ovarian cancer have unsatisfactory results. KEGG signaling pathway was enriched into multiple metabolic pathways, and these biological processes were related to AKT-mTOR signaling pathway. High expression of TTK inhibited cell apoptosis, thereby leading to tumor enlargement. Furthermore, the TTK was not only the hub biomarker of ovarian cancer, but also one significant hub gene of renal cancer, and its expression was up-regulated in the renal cancer. Compared with the renal cancer patients with low expression of TTK, the patients with high expression of TTK have the poor overall survival (*P* = 0.0021).

Ovarian cancer and renal cancer are prone to invasive growth and metastasis [12]. Nowadays, ovarian cancer and renal cancer group in China are gradually younger, urban women are more susceptible to disease. Ovarian cancer and renal cancer are of great harm, which not only has a great impact on the patient's physiology and psychology, but also has a high treatment cost, which causes a heavy burden on the patient's family economy.

TTK is a bispecific protein kinase [13, 14]. When it makes too many centrosomes, it has potential to trigger tumors, affecting mitotic spindles [15]. TTK functions in relation to cell proliferation and enhances Aurora kinase B activity [16]. TTK is a regulator of cell cycle, [17], and is linked to tumorigenesis [18]. TTK can help bladder cancer cells activity and mediate epithelial-mesenchymal transition [19]. There is evidence that TTK linked to glioblastoma [20]. Elevated TTK levels lead to centrosome enlargement and chromosomal instability, which leads to tumorigenesis [21, 22]. Therefore, it is possible that TTK has a certain effect on ovarian cancer and renal cancer.

AKT belongs to AGC family of protein kinases [23]. Activity of AKT affects cell function [24], activate protein translation and enhance cell growth, phosphorylates target proteins in cytoplasm and nucleus [25, 26], stimulates cell reproduction [27]. It has been shown that inhibition of Akt can affect tumor cells [28]. Other studies have shown that, AKT is a therapeutic target for cancer [29]. AKT can directly phosphorylate mTOR and act indirectly on mTOR.

The mTOR is the serine/threonine protein kinase [30]. And it acts on signaling pathway of cell reproduction [31], and is influenced by cell signaling [32]. In cells, it exists in form of two different multiprotein complexes. One is mTORC1, it stimulates cell development and is activated primarily via PI3P/AKT pathway [33]. One is mTORC2, it promotes AKT activation through direct phosphorylation of its hydrophobic motif (Ser473) [34]. mTOR kinases are involved in key events that integrate external and internal signals, coordinating cell growth and proliferation. Multiple components of the signaling pathway that signals through mTOR are dysregulated in many cancer types. Therefore, mTOR can be a good entry point for tumor treatment [35].

Akt/mTOR is important signaling pathway of cellular activity, which can regulate cell size, metabolism, motility, so on [36]. PI3K/AKT/mTOR pathway affects normal cellular processes, also have abnormal manifestations in many cancers [37]. It is evidence that, PI3K/Akt/mTOR pathway targets non-small cell lung cancer [38], also affects breast and gastric cancer [37, 39]. Akt/mTOR pathway may be influenced by many factors, its activation has been implicated in pathogenesis of a variety of tumors [40]. Thus, we hypothesized, AKT-mTOR pathway can affect cancer. TTK can affect cancer cells through AKT-mTOR pathway [41]. This supports our point, TTK inhibits apoptosis through AKT-mTOR pathway, which in turn leads to worsening of cancer.

Our investigation also has some shortcomings, we have not conducted clinical validation to solidify this view. We should explore this more next.

In summary, TTK and AKT-mTOR pathways affect ovarian cancer. High TTK expression means poor outcomes for ovarian cancer patients. And TTK was also one significant hub biomarker of renal cancer.
